# Modeling inactivation of non-proteolytic *Clostridium botulinum* type B spores in a plant-based fish alternative

**DOI:** 10.3389/fmicb.2024.1509681

**Published:** 2024-12-18

**Authors:** Chrysanthi Champidou, Mariem Ellouze, Nabila Haddad, Jeanne-Marie Membré

**Affiliations:** ^1^Food Safety Research Department, Nestlé Research, Lausanne, Switzerland; ^2^Oniris VetAgroBio, INRAE, SECALIM, Nantes, France; ^3^Digital Food Safety Department, Nestlé Research, Lausanne, Switzerland

**Keywords:** group II *Clostridium botulinum*, vacuum-packed chilled products, REPFEDs, pasteurization, predictive microbiology

## Abstract

Our study aims to assess the thermal inactivation of non-proteolytic type B *Clostridium botulinum* spores in a plant-based fish and to evaluate the potential of alternative heat treatments at temperatures below the safe harbor guidelines established for vacuum-packed chilled products of extended durability. First, the heat resistance of the spore suspension was determined using capillary tubes in potassium phosphate buffer at 80°C. The D_80_ value was estimated to be 0.7–0.8 min. Then, inactivation was studied in a plant-based fish alternative using “thermal cells equipment.” Inactivation kinetics were obtained at four temperatures: 78, 81, 84 and 85°C, in duplicates. A secondary model describing log_10_D values versus temperatures was fitted to the dataset. The model parameters Z_T_ and log_10_D_ref_ (log_10_D at T_ref_ 82°C) were estimated to be 8.02 ± 0.46°C and 0.32 ± 0.02, respectively. Model validation was done first with additional data collected at three different temperatures (79.1, 82.5, 87.5°C) and second with literature data. The time required to deliver 6 log reduction in the plant-based food matrix was predicted at temperatures within the range 80–90°C. The recommended processing for vacuum-packed chilled products, 90°C for 10 min, was evaluated. We demonstrated that the recommended processing is approximately five times more than the time required for 6 log reduction of non-proteolytic *C. botulinum* in the plant-based fish alternative, indicating a substantial margin of safety. Our findings highlight the importance of conducting product-specific studies for the evaluation of thermal processing and the potential of process optimization for certain product categories.

## Highlights

- Non-proteolytic *Clostridium botulinum* inactivation model in plant-based fish.
- Model validation performed at three different temperatures.
- Z_T_ value was 8.02°C and log_10_D_82°C_ 0.32 in the plant-based fish.
- Time for 6D at 90°C was estimated to be 1.26 min in the plant-based fish.
- Time/temperature combinations for 6D within 80–90°C were predicted.

## 1 Introduction

Among the four groups of *Clostridium botulinum*, Groups I and II are the most studied ones, due to their link with foodborne botulism (Carter and Peck, 2015). Group I proteolytic clostridia are known for high heat resistance and have set the basis for the food safety objective linked to sterilization, also known as “*Botulinum* Cook” or F_0_ = 3 min in the food industry (Bean et al., 2012).

Non-proteolytic *C. botulinum* can grow at low temperatures but do not exhibit as high heat resistance as proteolytic *C. botulinum*, so the pasteurization at 90°C for 10 min can eliminate their spores. This is usually referred to as a safe harbor. However, in case of insufficient treatment or in case of recontamination, Group II strains can pose a risk during chilled shelf-life storage, since they are psychrotrophic with a wide growth range, 3.3–45°C (Peck et al., 2011). Moreover, once they multiply, they are able to produce neurotoxin even at temperatures as low as 3.3°C, although slowly (Advisory Committee on the Microbiological Safety of Food, 2020; European Chilled Food Federation, 2006). According to Peck et al. (1995), as little as 25–50 ng of botulinum neurotoxin (BoNT) is enough dose to cause illness or death.

These extreme growth limits have established non-proteolytic *C. botulinum* as the target organism for thermal processing of refrigerated foods with extended durability (REPFEDs). According to the Advisory Committee on the Microbiological Safety of Food (ACMSF), there are certain controlling factors recommended to prevent growth and toxin production of non-proteolytic *C. botulinum* in this product category. These include a pasteurization at 90°C for 10 min (Advisory Committee on the Microbiological Safety of Food, 2020; European Chilled Food Federation, 2006), which is widely used by Food Business Operators worldwide.

In the absence of product-specific recommendations, plant-based foods, intended to be stored at chilled conditions with an extended durability, fall under the framework of this processing recommendation. However, it is important to explore the possibility of less intense heat treatments, that could potentially allow improvement of the sensory profile of newly developed food products, such as plant-based meat or fish alternatives.

In general, low presence of the organism is expected in raw materials and it is considered difficult to detect (Barker et al., 2016). However, presence has been reported in vegetables (Lindström et al., 2006), mushrooms (Barker et al., 2016), as well as thickening agents (Carlin et al., 2004). In addition, Carlin et al. (2000) mentioned that within the period 1969–1989 more than 1,000 outbreaks linked to products containing or highly probable to contain vegetables have been reported due to *C. botulinum* contamination. More recently, Pernu et al. (2020) reported high prevalence of non-proteolytic *C. botulinum* in vegetarian sausages.

Although the thermal processing of non-proteolytic *C. botulinum* has been studied in multiple heating media and food matrices (Wachnicka et al., 2016) there are still no published data pertinent to plant-based foods. The objective of the present study was to provide data contributing to the filling of this knowledge gap by:

- collecting data and modeling the thermal inactivation of non-proteolytic type B *C. botulinum* spores in a plant-based food matrix, and
- investigating the potential of alternative milder thermal processing conditions, compared to the 90°C/10 min heat treatment, to ensure the safety of plant-based matrices with regards to non-proteolytic *C. botulinum.*

## 2 Materials and methods

### 2.1 Plant-based food matrix

Inactivation of non-proteolytic *C. botulinum* was studied in a plant-based fish alternative. The food product was selected as a model plant-based food matrix that, according to international guidelines (Advisory Committee on the Microbiological Safety of Food, 2020; European Chilled Food Federation, 2006), has to undergo pasteurization at 90°C for 10 min to achieve a minimum of 6 log reduction of non-proteolytic *C. botulinum*.

The product is composed of pea-protein, water, oil, salt and flavors. The pH was measured with a SevenMulti pHmeter (Mettler Toledo, Switzerland) and a probe for solid surfaces and the water activity (a_w_) with an Aqualab 4TE (METER Group, Germany). The product’s pH and a_w_ at 25°C was 5.6 0.985.

### 2.2 *Clostridium botulinum* spores

A Group II type B strain was selected as type B are psychrotrophic, having a T_min_ 2.5 and 3.0°C for growth and toxin production, respectively (Peck et al., 2011), so this makes them a potential hazard for products with chilled shelf-life. In addition, the processing guideline recommended for such products is based on heat resistance data obtained with a type B strain, so to ease comparison the same type was chosen.

Spores of the non-proteolytic Group II *C. botulinum* type B strain 3,908, corresponding to a clinical isolate, were purchased from miprolab (Germany), that prepared the spore suspension following their internal sporulation protocol. The spores were suspended in sterile distilled water and stored at 4°C until usage.

The concentration of the spores was verified with and without heat treatment at 65°C for 30 min of aliquots of the spore suspension, to evaluate the potential presence of vegetative cells. In addition, the heat resistance of the spores was tested in potassium phosphate buffer 10 mM (pH 7.0) at 80°C. The heat resistance control was performed with the methodology of capillary tubes as previously described in Champidou et al. (2024), modified to 50 μL glass bulbs (R&L Slaughter, United Kingdom) instead of 100 μL tubes.

### 2.3 Inactivation experiments

Inactivation in the food matrix was studied at four temperature levels: 78, 81, 84and 85°C (targeted levels, measured values shown in Table 1). Experiments were conducted in duplicates for the model development and additional kinetics were generated at 79.1, 82.5 and 87.5°C (targeted levels: 79, 83 and 87°C) for the model validation. For each of the targeted levels, the water bath was set to 1–2°C higher. The temperature range of the study was set based on the spores’ heat resistance, to allow for proper sampling during heating and to test temperatures close to the reference thermal processing for such foods.

**Table 1:** Primary inactivation parameters with standard errors (SE) and relative standard errors (RSE) of non-proteolytic *C. botulinum* type B 3908 in plant-based fish alternative and fittings’ Mean Square Error (MSE).

Temperature (°C)	Replicate	Log_10_N_0_ (CFU/g) ± SE	Log_10_N_0_ RSE	k_max_ (min^−1^) ± SE	k_max_ RSE	D (min)	MSE
78.41	A	6.35 ± 0.27	4%	0.34 ± 0.04	12%	6.78	0.483
78.11	B	5.73 ± 0.31	5%	0.37 ± 0.04	11%	6.22	0.303
80.83	A	6.63 ± 0.23	3%	0.89 ± 0.07	8%	2.60	0.338
80.93	B	6.51 ± 0.49	8%	0.93 ± 0.14	15%	2.48	0.585
83.50	A	5.42 ± 0.24	4%	1.57 ± 0.19	12%	1.47	0.177
83.84	B	5.11 ± 0.19	4%	1.69 ± 0.13	8%	1.36	0.103
84.60	A	6.35 ± 0.36	6%	2.65 ± 0.35	13%	0.87	0.593
85.53	B	5.38 ± 0.38	7%	2.87 ± 0.49	17%	0.80	0.554

The heat treatment was performed with the use of “thermal cells” equipment, which was designed specifically for heat treatment of solid food matrices, similar to the approach proposed by Chung et al. (2008).

#### 2.3.1 Thermal cell design and heat treatment

**Figure 1:**
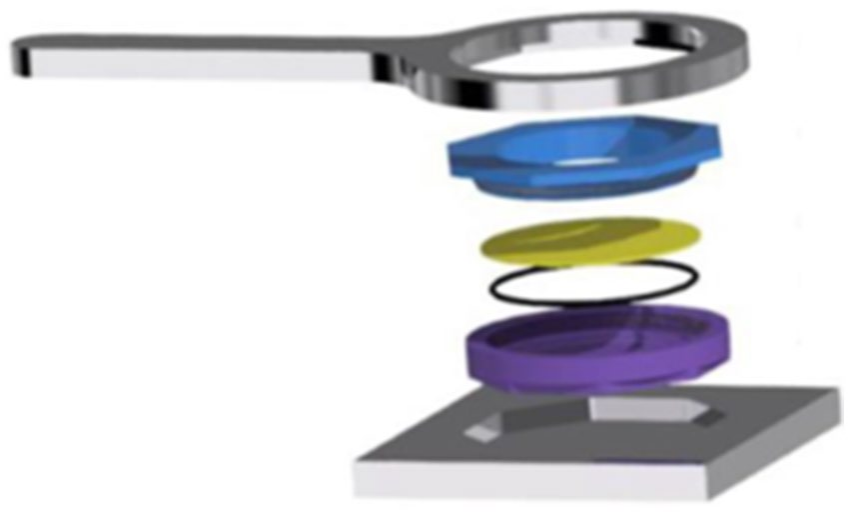
Design of a thermal cell (blue, yellow, purple and black parts) and its screwing equipment (silver parts).

“Thermal cell” design consists of three parts, the base (purple), the lid (blue) and an intermediate disk (yellow). Figure 1 illustrates the design of a thermal cell and screwing equipment. All parts are made of aluminum to allow for high conductivity, (i.e., between 170 and 202 W/mK for the base and lid and 205 W/mK) for the k All materials have been treated with hard anodic oxidation, preventing aluminum corrosion and improving the mechanical properties.

Before the treatment, 1 g of food matrix was placed in the cavity of the cell base (purple part in Figure 1). The matrix was artificially inoculated by pipeting 0.1 mL of 9 log_10_ CFU/mL spore suspension and dispensing this volume in five individual spots of the surface of the food sample, resulting in an initial contamination of 7 log_10_ CFU/g, approximately. Once inoculated, the matrix was covered by the aluminum disk and the thermal cell lid was, subsequently, screwed hermetically to the base cavity.

For each heat treatment, approximately 8–10 thermal cells were prepared and placed simultaneously into a temperature-controlled water bath Clifton Range Nickel-Electro LTD bath (VWR International GmbH, Switzerland). The core temperature during the heat treatment was electronically monitored with the TC-08 thermocouple data logger, a PTFE insulated type k thermoprobe (Pico Technology, United Kingdom) inside a thermal cell. Temperature data were obtained with 1-s interval and analyzed with the use of the Picolog® software (Pico Technology, United Kingdom).

The first sampling point (t_0_) was performed when the thermal cell equipped with the thermoprobe reached the targeted core temperature, while tolerance of ±0.3°C was applied. At each pre-defined time point, one thermal cell was removed from the bath and immediately immersed in an ice-bath to rapidly stop the inactivation before enumeration.

#### 2.3.2 Sample analysis

After the heat treatment, thermal cells were opened and samples were transferred into 80 mL Stomacher Strainer Closure bags (Seward, United Kingdom). Then, a decimal dilution was performed in Dilumat (bioMérieux, France) by adding 9 mL of physiological salt solution (TS; in house preparation: tryptone, Oxoid™ and NaCl, Merck, Switzerland) in each bag containing 1 g heat-treated food matrix. The samples were homogenized for 2 min in a Stomacher Sample Blender (bioMérieux, France) set at “normal” speed. Serial 1:10 dilutions were performed in TS and 1 mL of the selected dilutions was pour plated in Reinforced Clostridial Medium (RCM) agar (Oxoid™, United Kingdom). Plates were incubated for 72 h at 30°C under anaerobic conditions. For the generation of anaerobic environment, anaerobic generator bags and indicators (Thermo Scientific™ Oxoid™, Switzerland) were used, while incubation was performed inside anaerobic jars (bioMérieux, France) sealed hermetically.

### 2.4 Modeling

For the model development, four temperature levels were tested in duplicates: 78.2 ± 0.21, 80.88 + 0.07, 83.67 ± 0.24 and 85.07 ± 0.65°C. For the validation of the model, three temperature levels 79.1, 82.5 and 87.5°C were tested without replicate. In total, 11 inactivation kinetics were generated.

Primary fittings were performed with the log linear regression model, shown in Equation 1, using the GinaFit software (Geeraerd et al., 2005).


(1) $$\log_{10}N=\log_{10}N_{0}-\frac{k_{\text{max}}\times t}{\ln\left(10\right)}$$

where *N* is the concentration of microorganisms, in log_10_CFU/g, surviving at time *t,* in min, *N*_0_ is the initial microbial concentration, in log CFU/mL, and *k*_max_ is the maximum specific decay rate, in min ^−1^. The time (min) required for 90% reduction of the microbial load, D-value, was derived based on the following Equation 2:


(2) $$D=\frac{1}{k_{\text{max}}}\times \ln\left(10\right)$$

A secondary model describing log_10_D values versus temperatures was fitted to the dataset (Equation 3):


(3) $$\log_{10}D=\log_{10}D_{ref}+\left(\frac{T_{ref}-T}{Z_{T}}\right)$$

where D_ref_ is the time required for 90% reduction of the microbial load at a reference temperature, T_ref_, and Z_T_ is the temperature increase required for 1 log reduction of the D-value. T_ref_ was set to 82°C, representing the average value of the measured temperatures.

The upper and lower 95% confidence and prediction intervals of the secondary model were calculated with two-tailed Student’s t test.

Model goodness-of-fit was evaluated based on the Mean Square Error (MSE), coefficient of determination (*R*^2^) and by checking visually the absence of bias in the plots presenting fitted values (Equation 3) versus estimated values derived from (Equation 2) after a log_10_ transformation.

The Relative Standard Errors (RSE) of the primary and secondary parameters were calculated as the percentage of the ratio of the standard error to the respective parameter estimate.

#### 2.4.1 Validation

To assess the extent to which the model can be applied, a double validation was conducted. First, the model was validated in the same matrices with the same spore suspension by comparing the predicted versus observed kinetics at static conditions at three temperatures: 79.1, 82.5 and 87.5°C. A combination of Equations 1–3, was used to predict the log_10_D values at the three target temperatures and inactivation over time was simulated. The log_10_N_0_ was fixed to the observed data at t_0_ and set to 6.63, 7.01 and 3.40 for 79.1, 82.5 and 87.5°C, respectively.

In addition, external validation of the results obtained in our study was carried out with published data on inactivation of non-proteolytic *C. botulinum* type B strains in both laboratory media (Juneja et al., 1995; Stringer et al., 1999; Wachnicka et al., 2016) and foods (Gaze and Brown, 1990; Juneja et al., 1995; Wachnicka et al., 2016). Out of these, data from studies where lysozyme was used were excluded, as it is known to affect the recovery of non-proteolytic *C. botulinum* spores. In total 135 published log_10_D values were plotted as function of temperature together with the model developed in our study.

Model performance was evaluated by estimating the bias (B_f_) and accuracy (A_f_) factors, as proposed by Ross (1996). The following equations, adjusted to inactivation models (Koseki and Yamamoto, 2007), were used:


(4) $$B_{f}=10^{\left(\sum \log\left(\log_{10}D_{\mathrm{pred}}/\log_{10}D_{obs}\right)/n\right)}$$

(5) $${Α}_{f}=10^{\left(\sum |\log\left(\log_{10}D_{\mathrm{pred}}/\log_{10}D_{obs}\right)|/n\right)}$$

where log_10_D_pred_ are the values predicted by the model, log_10_D_obs_ are the values observed experimentally and n is the number of observations used in the calculation.

With the literature dataset, when negative ratio of prediction versus observation was obtained, the value was excluded (since logarithm of a negative value cannot be computed); this resulted in 103 out of 135 log_10_D values used for the validation.

#### 2.4.2 Prediction of time/temperature combinations for 6-log reduction

Once the secondary model was developed and validated, it was used to predict *C. botulinum* inactivation in the range of 80–90°C to compare with the safe harbor of 90°C for 10 min for vacuum-packed products (Advisory Committee on the Microbiological Safety of Food, 2020; Bean et al., 2012; European Chilled Food Federation, 2006) and, also, investigate the effect of milder heat treatments. The estimation was performed with the following equation (Membré et al., 2007):


(6) HTT=PC×D

where HTT is the heat treatment time (min), D is the D value (min) and PC the performance criterion, in this case equal to 6, as the guideline recommendation is based on 6-log reduction during thermal processing.

The time required for 6-log reduction was predicted in some additional food matrices, based on data provided for different type B strains in literature (Gaze and Brown, 1990; Juneja et al., 1995; Wachnicka et al., 2016). The D values at 90°C, when not provided, were estimated with Equation 3 and the time for 6D at 90°C was simulated with Equation 6.

## 3 Results

### 3.1 Spores’heat resistance verification

The initial concentration of the spores was confirmed after heating at 65°C for 30 min to 9.28 log_10_ CFU/mL. To verify the heat resistance of the spores, a first assessment was performed to evaluate the D values at 80°C in potassium phosphate buffer.

**Figure 2:**
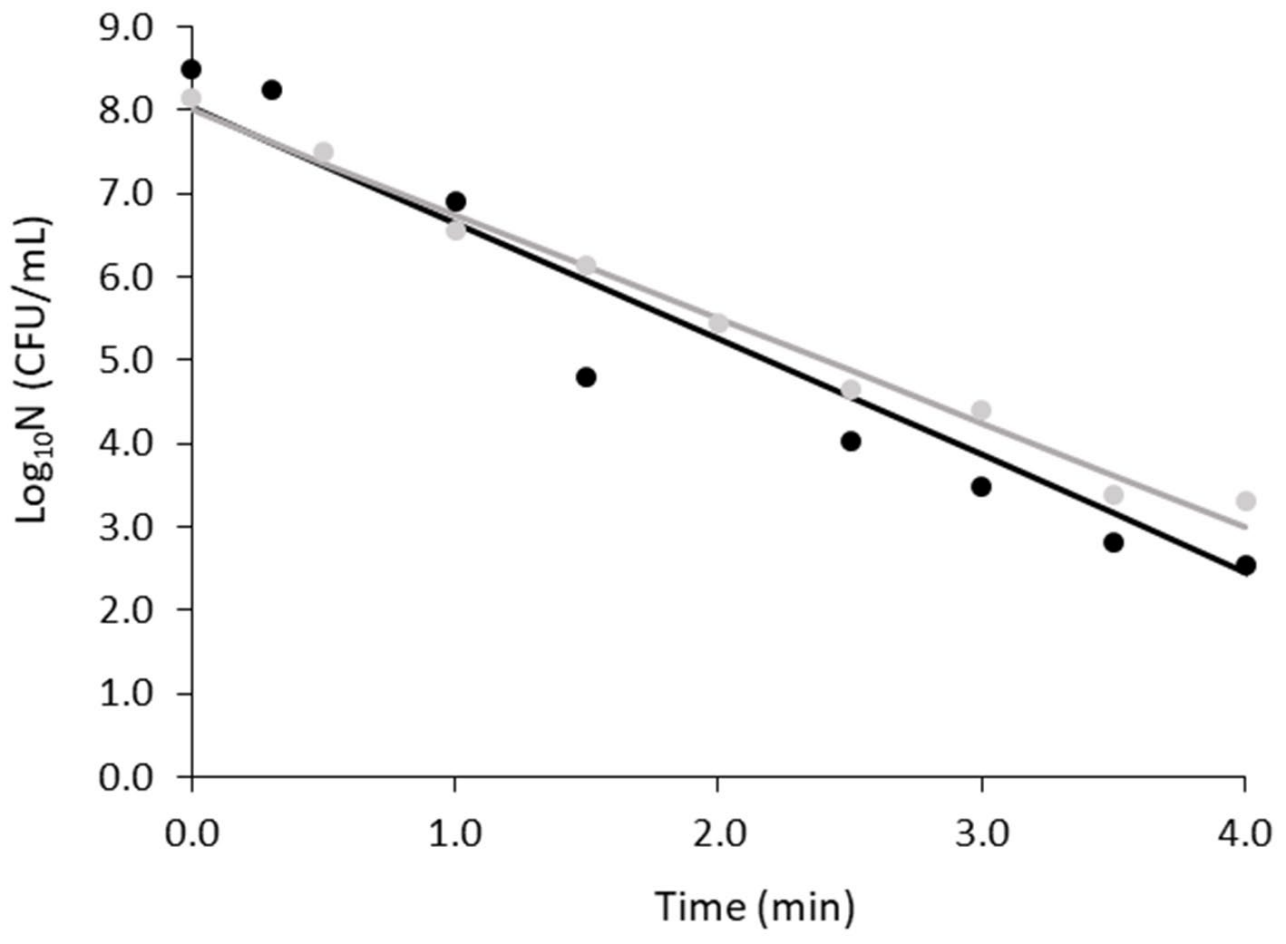
Fittings of non-proteolytic *C. botulinum* type B 3908 inactivation kinetics at 80°C in potassium phosphate buffer. Regression line (—), observations (replicate A ●, replicate B ●).

Figure 2 shows the fittings for the two replicates obtained at 80°C and Table 2 shows the primary model parameters, their standard errors, together with the overall MSE of the fits. Slight differences were observed in the shapes of the duplicates, with replicate B demonstrating a certain deviation from linearity compared to replicate A. This was reflected in the MSE of the fitting (Table 2). However, the RSE around the parameter estimation was low in both cases and the D values were similar, namely 0.8 and 0.7 min for replicates A and B, respectively. Therefore, the obtained fits were considered sufficient to verify the heat resistance of the spores, which was the purpose of this part of the study.

**Table 2:** Primary inactivation parameters with standard errors (SE) and relative standard errors (RSE) of non-proteolytic *C. botulinum* type B 3908 at 80°C in potassium phosphate buffer and fittings’ Mean Square Error (MSE).

Replicate	Log_10_N_0_ (CFU/g) ± SE	Log_10_N_0_ RSE	k_max_ (min^−1^) ± SE	k_max_ RSE	D (min)	MSE
A	8.00 ± 0.13	2%	2.88 ± 0.12	4%	0.80	0.041
B	8.15 ± 0.34	6%	3.27 ± 0.29	12%	0.70	0.451

The spores demonstrated moderate heat resistance, indicating that the tested strain is neither overly sensitive, nor extremely resistant. Therefore, it was considered as an average heat resistance scenario and inactivation was studied in the food matrix.

### 3.2 Heat inactivation model and parameters

**Figure 3:**
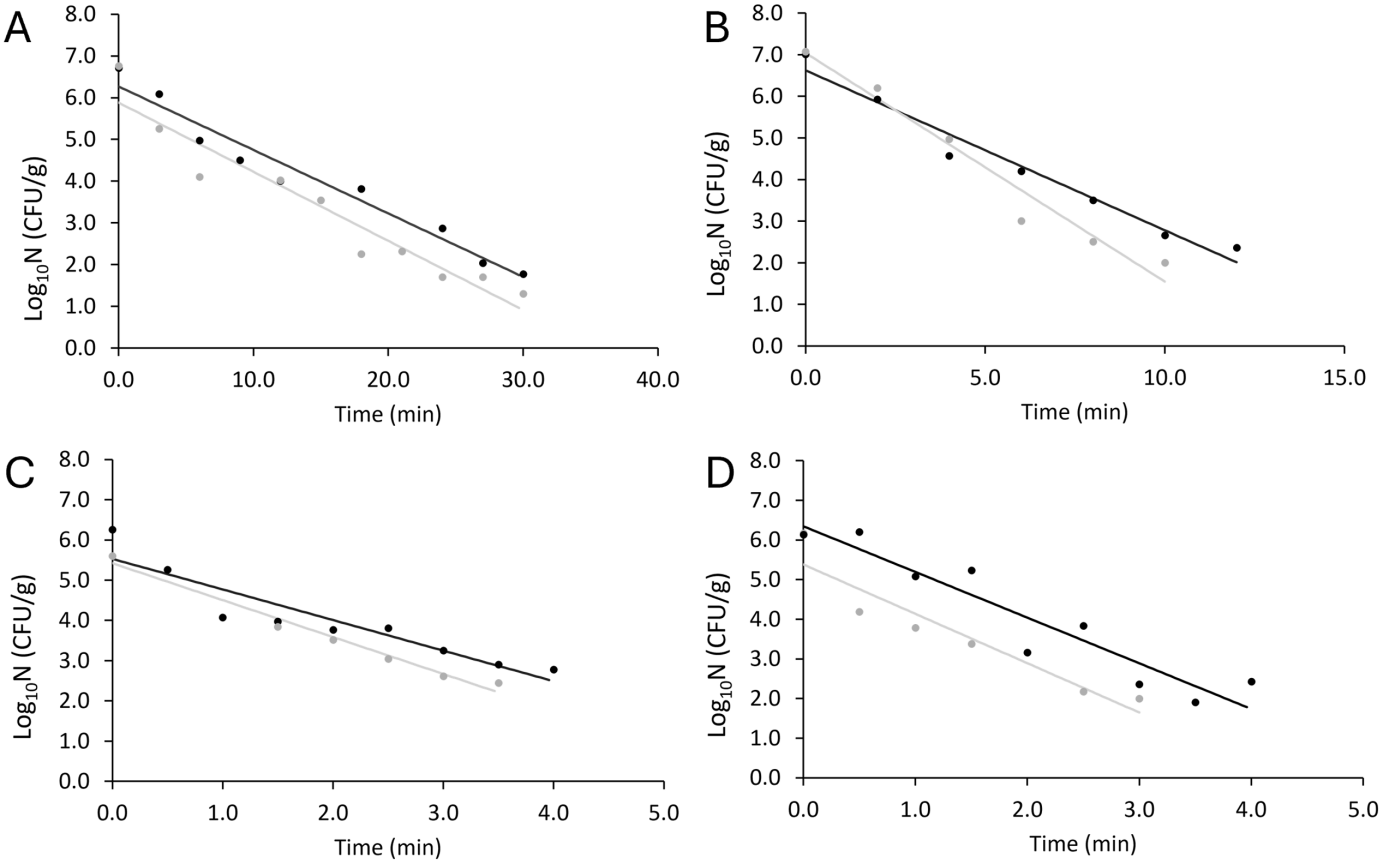
Fittings of non-proteolytic *C. botulinum* type B 3908 inactivation kinetics in plant-based fish alternative at 78°C **(A)**, 81°C **(B)**, 84°C **(C)** and 85°C **(D)**. Regression line (—), observations (replicate A ●, replicate B ●).

The data generated from the inactivation experiments were plotted against the heat treatment time to generate the inactivation kinetics in the food matrix. Figure 3 illustrates the two replicates obtained at the four tested temperature levels. The first time point, t_0_, represents the concentration after the target temperature was achieved, thus, at 84 and 85°C the t_0_ is slightly lower than at 78 and 81°C. The variability between the duplicate curves at 85°C is associated with the difference in the actual temperature reached in each replicate. All measured temperatures and model parameters are presented in Table 1.

The log linear model was fitted to the kinetics obtained. Table 1 shows the primary parameters estimated with Equation 1 and D values calculated with Equation 2 per temperature. Although a relative variability is captured in Figure 1, deviation between the D values was low, between 0.07 and 0.39 min.

The MSE was around 0.5 while the relative standard errors (RSE) of log_10_N_0_ and k_max_ were around 5 and 15%, respectively. At the highest temperature tested (85.5°C), the RSE values were 7 and 17%, respectively. It is expected that at higher temperatures the quality of the data is lower due to experimental challenges in sampling. However, since the two D value replicates were relatively similar, even at the highest temperature, the quality of the fit was considered adequate.

**Figure 4:**
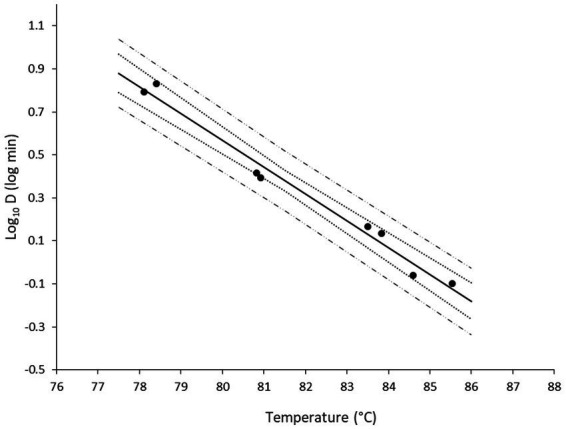
Secondary model describing the change in log_10_D of non-proteolytic *C. botulinum* type B 3908 in the plant-based fish alternative as function of temperature. Regression line (—), observations (●), 95% confidence intervals (∙∙∙), 95% prediction intervals (− −).

Once the D values were obtained, a secondary model was developed with the log transformation of D as function of temperature (Figure 4). The variability between the biological replicates was low. Note that there were slight temperature differences between the experiments that explain why the two replicates are not always aligned on an identical temperature level. The 95% confidence intervals of the regression were narrow, illustrating more robust model performance toward the middle of the studied temperature range.

**Figure 5:**
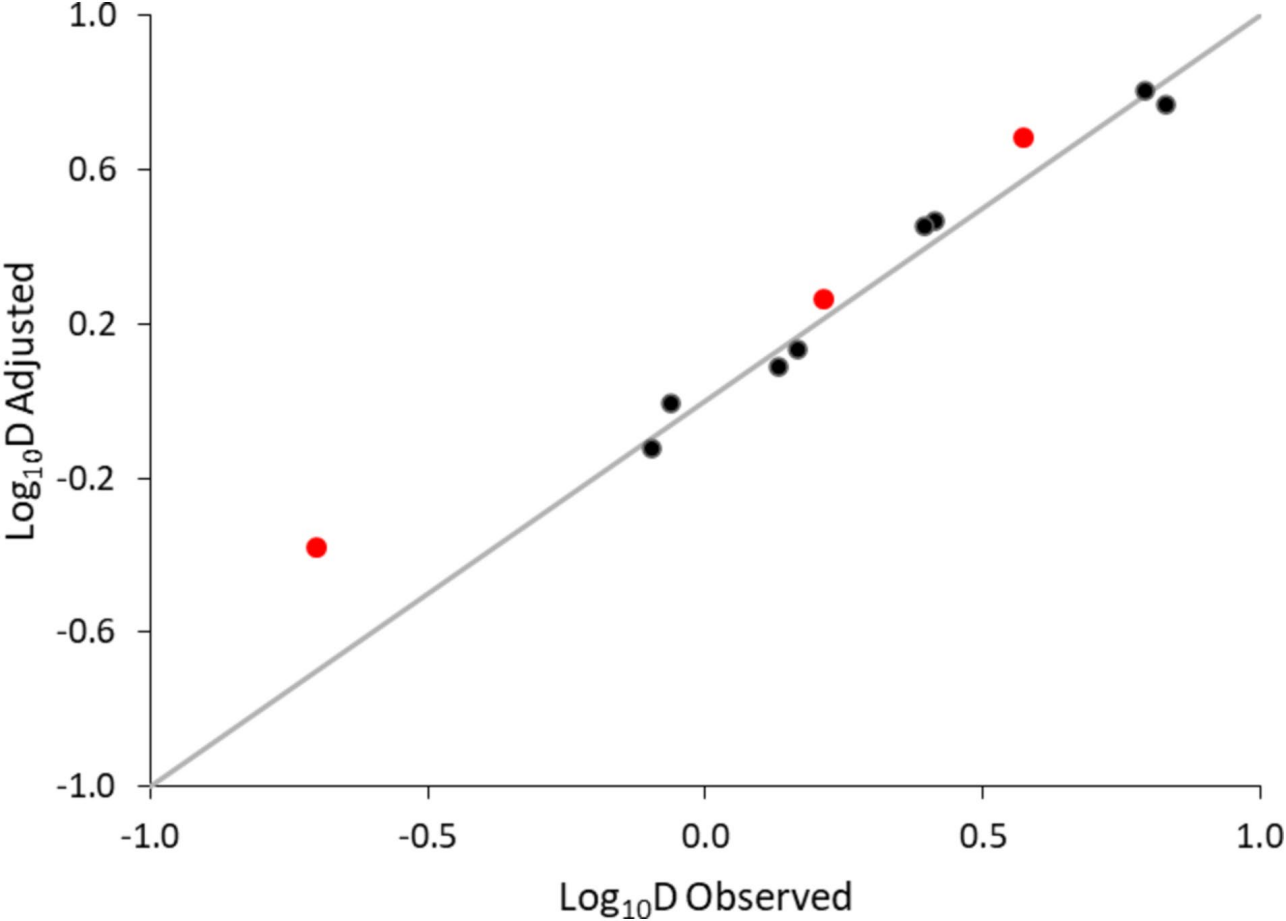
Adjusted versus observed log_10_D values of non-proteolytic *C. botulinum* type B in the plant-based fish alternative. Data used for model development (●), data used for validation using additionally generated data (●) and equality line (—).

Table 3 provides the inactivation parameters with their standard errors and the coefficient of determination. With RSE values of 6% and *R*^2^ of 0.98, the secondary model was considered accurate. Moreover, the model predictions were plotted against the observations. Figure 5 shows an overall satisfactory agreement between the adjusted and observed values, with no apparent bias and good homogeneity of variance.

**Table 3:** Secondary inactivation parameters, Z_T_ (°C) and log_10_D_ref_ (log min) with standard errors (SE) and relative standard errors (RSE) of non-proteolytic *C. botulinum* type B 3908 in the plant-based food matrix.

Z_T_ ± SE	Z_T_ RSE	Log_10_D_ref_ ± SE	Log_10_D_ref_ RSE	*R* ^2^
8.02 ± 0.46	6%	0.32 ± 0.02	6%	0.980

T_ref_ was set to 82°C. *R*^2^ stands for regression coefficient.

### 3.3 Validation

**Figure 6:**
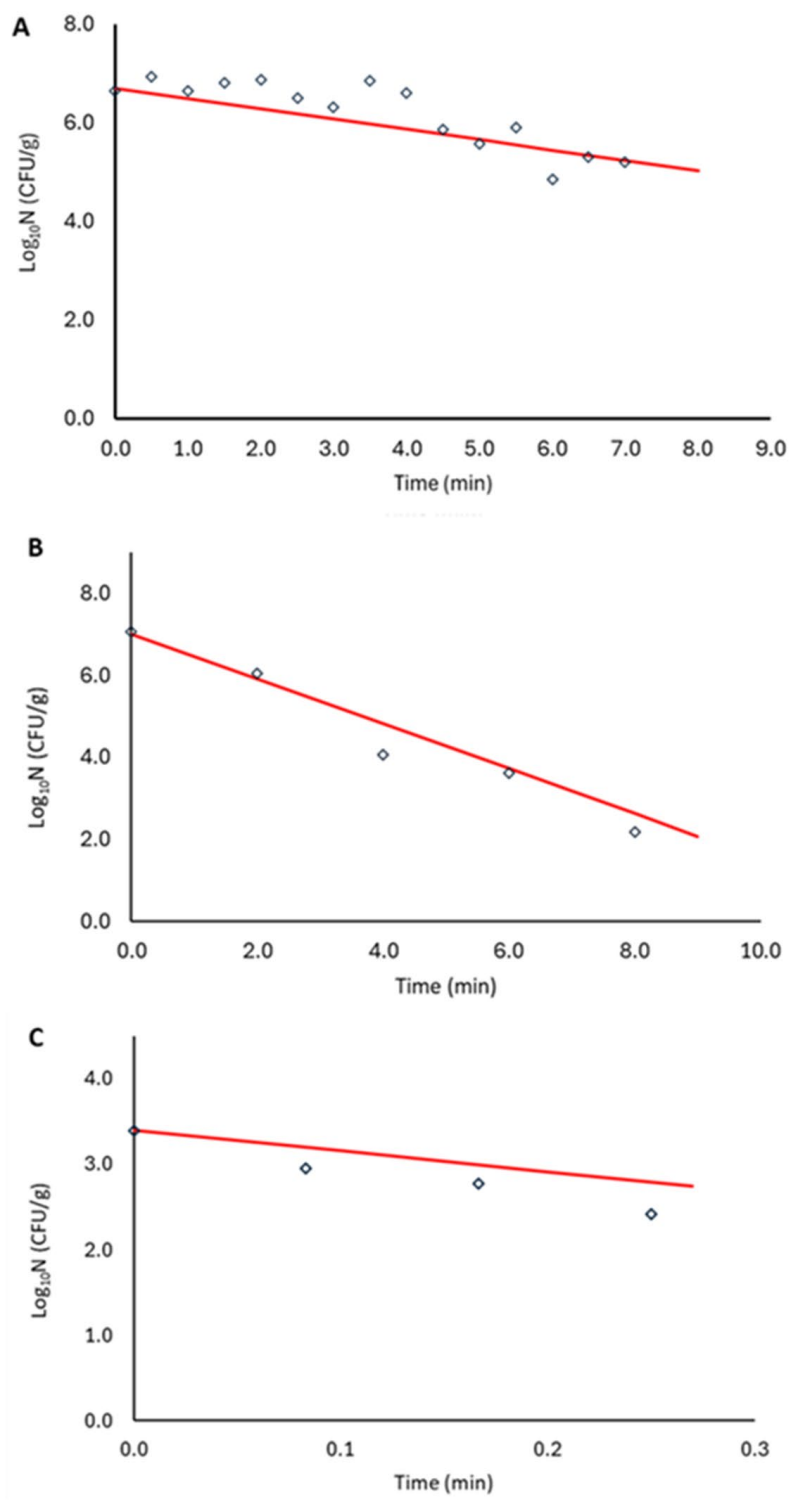
Non-proteolytic *C. botulinum* type B 3908 model validation in plant-based fish alternative at 79.1°C **(A)**, 82.5°C **(B)** and 87.5°C **(C)**. Model predictions (—), observations (○).

Model validation was performed in two steps. First, log_10_D values generated at 79.1, 82.5 and 87.5°C were used for validation (red mark in Figure 5). The bias and accuracy factors were 0.92 and 1.38, respectively. In addition, the kinetics predicted by the secondary model were compared with the observed data in the plant-based food matrix (Figure 6). In Figure 6, red lines correspond to predictions, while marker points to experimental observations at pre-defined time points. At 87.5°C where inoculum was lower due to increased temperature, Equation 1 was adjusted with the respective log_10_N_0_ level. In all scenarios the model showed satisfactory performance, as the difference between the observed and predicted values was less than 1 log_10_ CFU/g.

**Figure 7:**
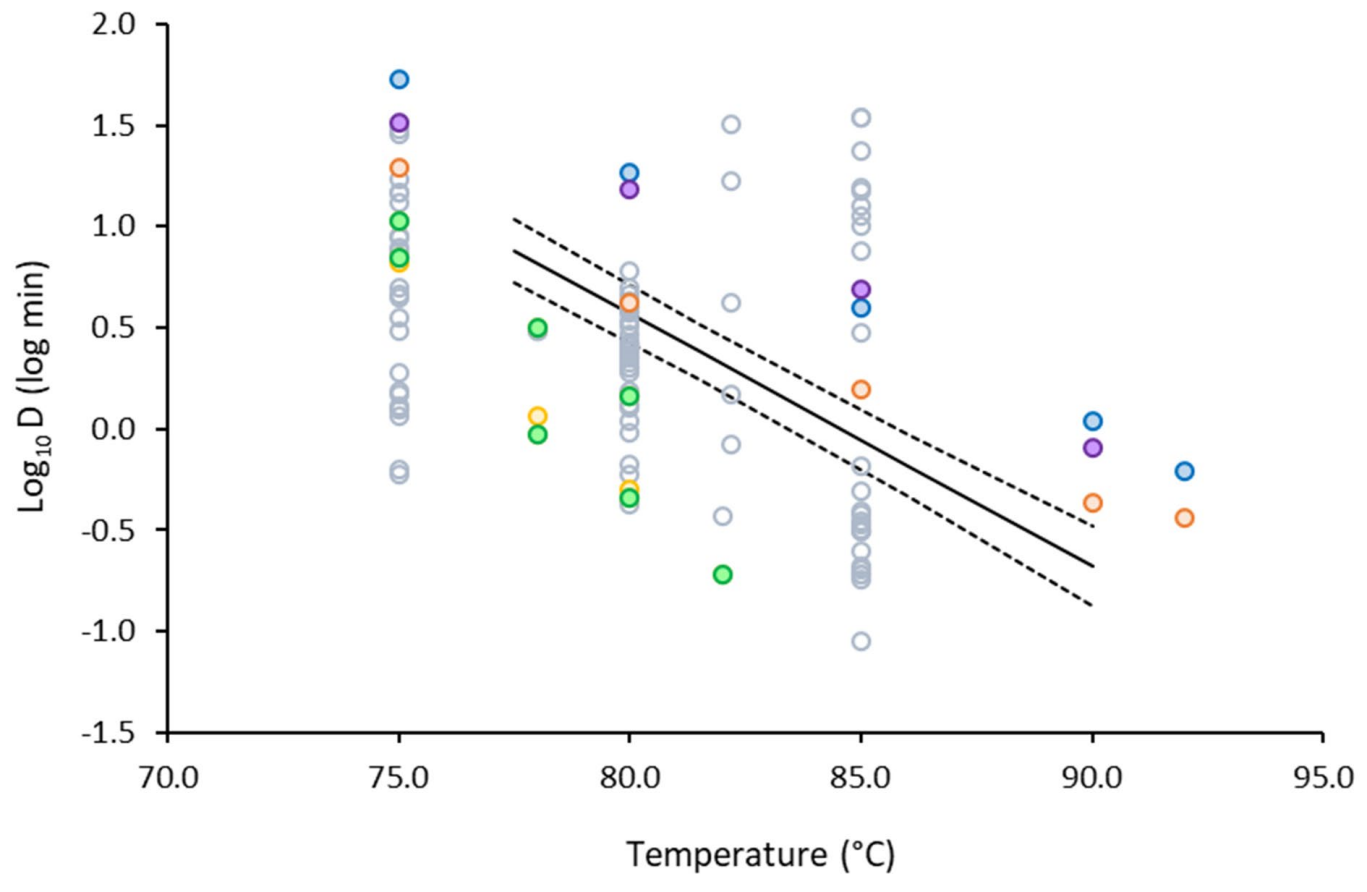
Change in the log_10_D values of non-proteolytic *C. botulinum* type B as function of temperature in: laboratory media (○) (Juneja et al., 1995; Peck et al., 1992; Scott and Bernard, 1982; Stringer and Peck, 1996; Stringer and Peck, 1997; Stringer et al., 1999; Wachnicka et al., 2016), potato puree (●), broccoli puree (●) (Wachnicka et al., 2016), carrot homogenate (●), cod fish (●) (Gaze and Brown, 1990), turkey slurry (●) (Juneja et al., 1995) and the model developed in our study (—).

To further validate our results, the data obtained in the plant-based matrix were plotted against published data for non-proteolytic type B strains (Figure 7). The developed model was validated against the literature data, using bias (Equation 4) and accuracy factors (Equation 5). Our model was well-aligned with literature: the B_f_ was estimated to be 1.16, which showed 16% deviation of predictions from observations. The A_f_ was estimated to be 2.61, which was not surprising as Figure 7 depicts a cloud of data obtained in various matrices, including laboratory media and foods, with pH ranging between 3.5 and 7.0.

The colored markers show a more refined selection of log_10_D values only in food matrices, with similar pH to that of the plant-based fish alternative and recovery of viable counts without the addition of lysozyme in the media. Green, yellow and orange markers, represent data in vegetable-based matrices, blue and purple in meat and fish and black lines the model developed in our study. Diverse range of heat resistance was reported in food matrices for the various strains tested. For example, log_10_D values ranged from −0.32 to 1.26 at 80°C, whereas in our study it was 0.57. Results show that our study represents an average inactivation scenario in food matrices.

Our data are closer to the inactivation profile obtained in carrot homogenate, which is not surprising given the plant-based composition and the similar pH of the two matrices; 5.6 for the plant-based fish alternative and 5.7 reported for the carrot homogenate. Overall, it is evident that less time is required for inactivation of *C. botulinum* in the vegetables and vegetable-based foods than in the fish or meat-based products.

Based on these observations and the B_f_ values, the model validation was deemed acceptable.

### 3.4 Predicted time and temperature combinations for 6 log reduction

First, inactivation was predicted in the plant-based fish alterative with Equation 3 and Equation 6 at 90°C, to assess the time required to achieve 6 log reductions at 90°C using the specific model developed in this study for the fish alternative, and then compare this processing time to the safe harbor recommendations (10 min).

Table 4 shows the predicted log_10_D value at 90°C with the respective confidence and prediction intervals.

**Table 4:** Predicted log_10_D value (log min) of non-proteolytic *C. botulinum* type B 3908 spores in the plant-based fish alternative at 90°C with 95% confidence and prediction intervals.

Temperature	Log_10_D	95% confidence intervals upper/lower	95% prediction intervals upper/lower
90°C	−0.68	−0.53/–0.83	−0.48/–0.88

The D_90_ was derived (0.21 min), and thus, the time for 6-log reduction (6D) at 90°C was estimated to 1.26 min, revealing an important safety margin compared to the recommended 10-min treatment time. As a worst-case scenario, the same simulation was performed with the upper prediction interval too; the time to 6D was estimated to 1.98 min, confirming the large safety margin with the recommendation for this particular product.

Then, the times required for a selection of temperatures to deliver the targeted 6 log reductions of non-proteolytic *C. botulinum* were predicted (Table 5).

**Table 5:** Time and temperature combinations delivering 6 log reduction (6D) of non-proteolytic type B *C. botulinum* 3,908 in the plant-based fish alternative, based on the predicted D and the 95% Upper Prediction Limit (UPL) of the model.

Temperature (°C)	Time to 6D (min)	Time to 6D based on 95% UPL
80	22.17	30.76
81	16.64	22.92
82	12.49	17.16
83	9.37	12.91
84	7.03	9.77
85	5.28	7.42
86	3.96	5.66
87	2.97	4.33
88	2.23	3.33
89	1.68	2.56
90	1.26	1.98

**Figure 8:**
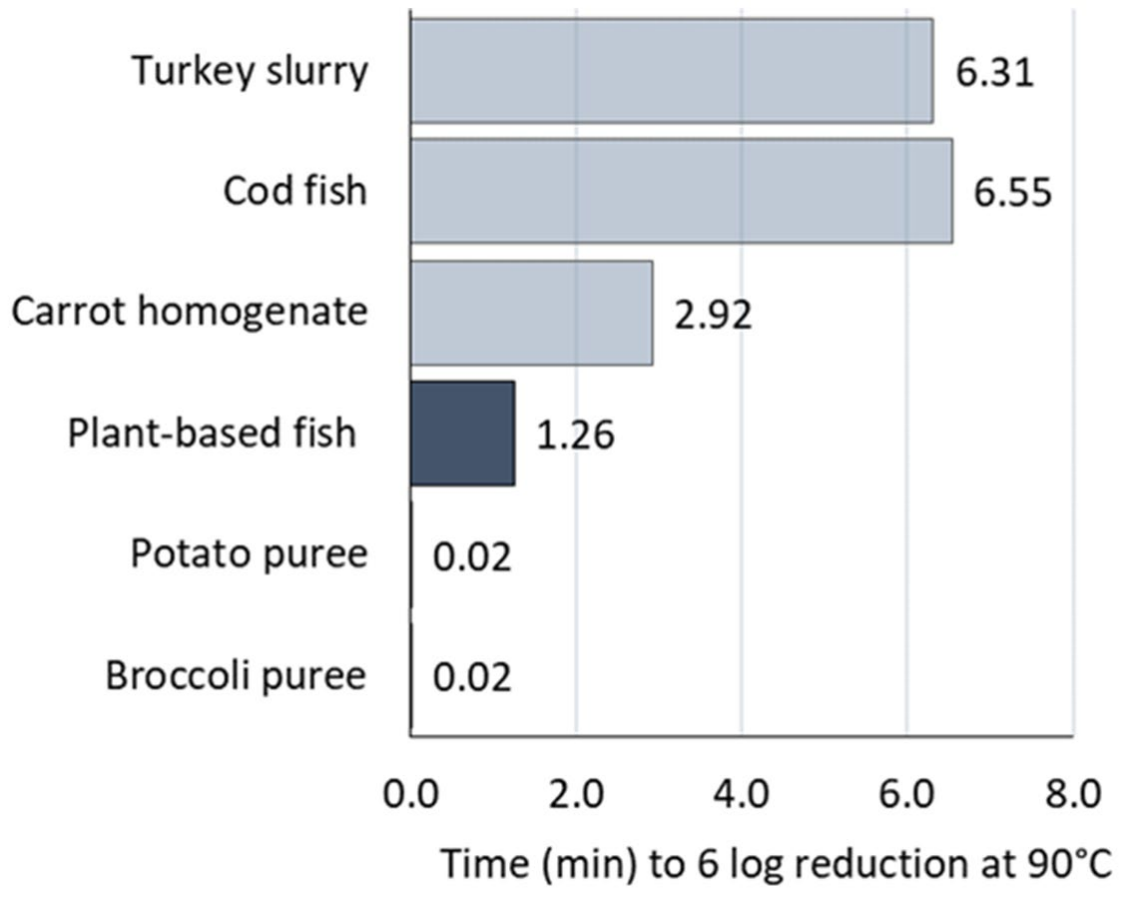
Heat treatment time (HTT) required for 6 log reduction at 90°C in the foods based on literature data (■) and the plant-based fish alternative of our study (■).

The estimated HTT was compared to published data for non-proteolytic *C. botulinum* type B strains inactivation in the food matrices that were shown in Figure 7. The secondary parameters of the literature datasets were estimated, when not provided, by modeling the log_10_D values given per temperature. Then, the heat treatment times were calculated with Equation 6 per food product (Figure 8).

Figure 8 shows clearly that the matrices can be distinguished in two sets of data: cod and turkey versus carrot on top, broccoli, potato and plant-based fish on the bottom. Inactivation up to 6 log reduction of *C. botulinum* 3,908 spores in the plant-based fish alternative tested in our study requires 1.26 min at 90°C or 1.98 min at worst-case. Less time is needed in broccoli and potato and double the time in carrot. On the contrary, longer times are required for turkey and cod, around 6.5 min.

## 4 Discussion

This study aimed to collect data and model the thermal inactivation of non-proteolytic type B *C. botulinum* spores in a plant-based food matrix. Moreover, our goal was to investigate the potential of alternative lower processing conditions compared to the 90°C/10 min heat treatment to ensure safety of plant-based matrices with regards to non-proteolytic *C. botulinum.*

To achieve this goal, first, we determined the heat resistance of the spores used in the study. The estimated D_80_ was 0.7–0.8 min in potassium phosphate buffer, which was in alignment with Juneja et al. (1995) who reported values within 0.6–1.9 min for spores originating from three different *C. botulinum* strains using the same buffer and recovery media as the ones used in our study. Once our spore crop’s thermal resistance was verified to be within the range previously reported in literature, inactivation was studied in the plant-based fish alternative.

Despite the experimental challenges commonly occurring in studies with anaerobic spore-formers, and solid matrices, linear inactivation patterns were observed in the food matrix. Besides, the variability between the two replicates per temperature was low. The latter was reflected in the estimation of primary parameters. The log_10_D values reported in the literature at 80°C ranged from −0.05 to 1.5 for different matrices (Wachnicka et al., 2016), while the log_10_D at 80°C based on our model was estimated to 0.57, therefore, approximately in the middle of the reported range. It is important to highlight that the above-mentioned values correspond to studies without the addition of lysozyme.

Wachnicka et al. (2016) demonstrated that in the absence of lysozyme the average Z_T_ value was 6.7°C, while in the presence of lysozyme in the recovery media it was 9.3°C for a variety of strains and matrices. In our study, where lysozyme was not added in the recovery media, Z_T_ was estimated to 8.02 ± 0.46°C. Since there are no published studies in inactivation of type B strains in plant-based products, we cannot directly compare our results.

However, several studies providing parameters of non-proteolytic *C. botulinum* inactivation have been published. Numerous studies have been conducted for type B strains in laboratory media, while only a few in food matrices. For example, Z_T_ values were within 4.60–5.68°C and 4.42–5.68°C in broccoli and potato puree, respectively, (Wachnicka et al., 2016) as well as 9.76, 8.60 and 9.39°C in carrot homogenate, cod fish and turkey slurry (Gaze and Brown, 1990; Juneja et al., 1995), respectively. Low values like those obtained in the vegetable purees are relatively unusual for bacterial spores, but the rest are in similar range with our estimate.

Next, we tested the performance of the model in the plant-based fish alternative at three additional temperatures. Validation showed satisfactory agreement between predictions versus observations. In addition, the model was validated against literature data which presented the advantage of using a large dataset (*n* = 135) and the disadvantage of mixing data from different strains, foods, experimental facilities, all of them sources of heterogeneity reflected in the accuracy factor (A_f_ = 2.61). Nevertheless, the bias factor was still rather low (B_f_ = 1.16). There are a few studies on spore-formers that have reported slightly higher values than the ones proposed by Mellefont et al. (2003) as acceptable. For example, B_f_ 1.21 in certain cases for *Bacillus cereus* (Juneja et al., 2024), A_f_ up to 1.35 for *Bacillus coagulans* (Zimmermann et al., 2014) and B_f_ 1.44 for *Bacillus subtilis* (Jagannath and Tsuchido, 2003) inactivation, but also for *C. botulinum* germination B_f_ between 0.61–1.13 and A_f_ 1.38–1.64 (Chea et al., 2000). Therefore, the validation was deemed acceptable.

Once validated, the developed model was used to simulate inactivation at 90°C, which is the reference processing temperature for vacuum-packed chilled foods with shelf-life longer than 10 days. The international guidelines propose pasteurization at 90°C for 10 min with the objective of achieving at least 6 log reduction (6D) of non-proteolytic *C. botulinum* (Advisory Committee on the Microbiological Safety of Food, 2020; European Chilled Food Federation, 2006). The predicted time for 6D at 90°C for the conditions tested in our study, i.e., food and strain, was 1.26 min (Table 5). As a worst-case scenario, if we would base the calculation on the upper prediction limit, the time for 6D at 90°C would be around 2 min, which is 5 times less than the safe harbor proposed application time. Generally, international guidelines like the 90°C / 10 min processing, always account for a margin of safety on top of the necessary processing conditions to ensure hazard elimination in a broad range of products and for a diverse spectrum of strains. Although our results concern only one strain, they indicate that there might be space for process optimization for products such as the plant-based fish alternative used in our study. Processing at milder temperatures could allow control of *C. botulinum* spores, while preserving sensory properties as the treatment intensity decreases. Based on the Degree of Protection (DoP) concept, a milder treatment combined with a specified shelf-life duration and chilled storage could provide sufficient confidence for the elimination of botulism hazard (Membré et al., 2009; Peck, 1997).

Therefore, in this study, we have provided a set of time and temperature combinations, below 90°C, delivering 6 log reduction (6D) of non-proteolytic *C. botulinum* in the plant-based product. In order to maintain realistic processing times and yet milder temperatures, we performed our assessment within the range 80–90°C. For example, at 85°C the time required for 6D in the plant-based fish alternative is 5.28 min or 7.42 min when considering the upper prediction limit as worst-case scenario. Similar approaches have been previously suggested by researchers targeting 6D reduction in other matrices (Fernandez and Peck, 1997; Membré et al., 2009; Peck et al., 1995; Peck, 1997).

Considering the constant demand for more sustainable solutions, alternative processing combinations shall be evaluated to support the implementation of new practices in the future. The current study constitutes a first assessment of the recommended processing on the inactivation of non-proteolytic *C. botulinum* type B 3908 spores in a plant-based fish alternative. The developed model can be used for risk assessments specific to plant-based meals, that so far, have been conducted with literature data on other matrices. Additional data in more food products and with different strains are required to draw robust conclusions and support potential optimization strategies in the established thermal processing regimes.

## 5 Conclusion

Our study provides a new model for the inactivation of spores of non-proteolytic *C. botulinum* type B in a plant-based fish alternative. We have provided a set of time and temperature combinations within the range 80–90°C delivering 6 log reduction in the plant-based food matrix.

The recommended processing for vacuum-packed chilled products, 90°C for 10 min, was evaluated. We demonstrated that the recommended processing enables sufficient control of non-proteolytic *C. botulinum* type B spores in the plant-based fish alternative with large margin of safety. The heat treatment time required for 6 log reduction at 90°C was estimated to 1.26 min for the product tested in our study.

Our findings highlight the importance of conducting product-specific studies for the evaluation of thermal processing and the potential of process optimization for certain product categories.

## Data Availability

The original contributions presented in the study are included in the article/supplementary material, further inquiries can be directed to the corresponding author.
